# On the Visuomotor Behavior of Amputees and Able-Bodied People During Grasping

**DOI:** 10.3389/fbioe.2019.00316

**Published:** 2019-11-15

**Authors:** Valentina Gregori, Matteo Cognolato, Gianluca Saetta, Manfredo Atzori, The MeganePro Consortium, Arjan Gijsberts

**Affiliations:** ^1^Department of Computer, Control, and Management Engineering, University of Rome La Sapienza, Rome, Italy; ^2^VANDAL Laboratory, Istituto Italiano di Tecnologia, Genoa, Italy; ^3^Information Systems Institute, University of Applied Sciences Western Switzerland (HES-SO Valais), Sierre, Switzerland; ^4^Rehabilitation Engineering Laboratory, Department of Health Sciences and Technology, ETH Zurich, Zurich, Switzerland; ^5^Department of Neurology, University Hospital of Zurich, Zurich, Switzerland

**Keywords:** visuomotor strategy, eye-hand coordination, upper-limb amputees, object segmentation, phantom limb movements, object tracking

## Abstract

Visual attention is often predictive for future actions in humans. In manipulation tasks, the eyes tend to fixate an object of interest even before the reach-to-grasp is initiated. Some recent studies have proposed to exploit this anticipatory gaze behavior to improve the control of dexterous upper limb prostheses. This requires a detailed understanding of visuomotor coordination to determine in which temporal window gaze may provide helpful information. In this paper, we verify and quantify the gaze and motor behavior of 14 transradial amputees who were asked to grasp and manipulate common household objects with their missing limb. For comparison, we also include data from 30 able-bodied subjects who executed the same protocol with their right arm. The dataset contains gaze, first person video, angular velocities of the head, and electromyography and accelerometry of the forearm. To analyze the large amount of video, we developed a procedure based on recent deep learning methods to automatically detect and segment all objects of interest. This allowed us to accurately determine the pixel distances between the gaze point, the target object, and the limb in each individual frame. Our analysis shows a clear coordination between the eyes and the limb in the reach-to-grasp phase, confirming that both intact and amputated subjects precede the grasp with their eyes by more than 500 ms. Furthermore, we note that the gaze behavior of amputees was remarkably similar to that of the able-bodied control group, despite their inability to physically manipulate the objects.

## 1. Introduction

Humans interact continuously with objects in activities of daily living (ADLs). Vision and gaze play an important role during these interactions, not only to guide the activity itself but also in the initial planning phase. Gaze is thus said to be anticipatory and can be used to understand an individual's intentions even before they manifest themselves in the motor domain. Several studies have attempted to explore this proactivity to help disabled people, such as in a robot assistant scenario (Admoni and Srinivasa, 2016; Koochaki and Najafizadeh, 2018; Saran et al., 2018). Another compelling use-case is the control of dexterous upper-limb prostheses (Castellini and Sandini, 2006; Markovic et al., 2014, 2015; Gigli et al., 2018), where deciphering the grasp intent from myoelectric activations alone can be challenging. The integration of gaze and vision as contextual information could be helpful especially during the initial transient phase of a movement. Executing this fusion successfully requires however a precise understanding of eye-hand coordination.

Gaze behavior has been studied extensively over the last decades. Early studies typically involved constrained settings, for instance by fixating the chin to avoid head movements or by limiting the field of view to a monitor (see Tatler et al., 2011, and references therein). Obviously, these findings may not be representative for unconstrained settings where free movement of the body is allowed to ensure natural behavior (Tatler et al., 2011; Tatler, 2014). Such unconstrained experiments became possible with the introduction of wearable eye-tracking devices that allowed the user to move freely in the environment (Land, 2006). Subsequently, studies on visuomotor coordination have confirmed that also in this setting actions are typically preceded by a visual fixation on the involved objects. This was verified during a block-copying task (Smeets et al., 1996; Pelz et al., 2001), while drinking from various objects (Belardinelli et al., 2016), during an object displacement task (Belardinelli et al., 2016; Lavoie et al., 2018), during pick-and-place of a bar (Johansson et al., 2001), and when grasping (Brouwer et al., 2009). Similar goal-oriented gaze strategies were also reported during ADLs, such as tea-making and sandwich-making (Land and Hayhoe, 2001), walking (Patla and Vickers, 2003), driving (Land and Lee, 1994), and sports (Land and McLeod, 2000; Hayhoe et al., 2012). Although all studies confirm the anticipatory nature of gaze, they do not always agree on the exact timing of the motor execution after the first visual fixation, for instance when the hand reaches the object. These discrepancies can probably be explained by differences in experimental setting (Smeets et al., 1996; Pelz et al., 2001), variability due to a small number of subjects, or difficulty in accurately analyzing a large number of trials.

Only a few studies have investigated the gaze behavior of amputees. In a small case study, Sobuh et al. (2014) observed that the amputated participants did not use gaze to proactively plan subsequent actions in a task. Instead, they tend to switch their gaze more often between the object and the prosthetic hand to visually monitor its proper functioning (Bouwsema et al., 2012; Hebert et al., 2019). This increased visual attention is most likely to compensate for the lack of tactile and proprioceptive feedback from their prostheses. A similar finding was also reported when able-bodied subjects were engaged in similar tasks using a prosthetic simulator (Blank et al., 2010; Sobuh et al., 2014; Parr et al., 2018, 2019). Almost all of these studies investigated this disruption in eye-hand coordination precisely for this reason, namely to measure the subject's proficiency in controlling the prosthesis. More visual attention to the hand area during reaching and manipulation is considered indicative of a lower level of skill and confidence in the control of the prosthesis. Conversely, it should therefore also be expected that gaze behavior will “normalize” with an increasing confidence in the control response of the prosthesis. Indeed, Chadwell et al. (2016) noted that one participant who used a prosthesis daily showed more natural gaze behavior than another less experienced participant, while Sobuh et al. (2014) observed a shorter fixation on the hand area with increasing practice.

In the present study, we investigate eye-hand coordination during reaching and grasping to determine the window of opportunity in which gaze can provide useful information for intent recognition. We used the data of the recently acquired dataset, in which 15 transradial amputees were asked to try to grasp and manipulate various household objects to the best of their ability with their missing limb. In addition, it contains data from 30 able-bodied control subjects who performed the same grasps and manipulation tasks with their right arm. Throughout the exercise, gaze, and visual data were recorded via eye-tracking glasses, while the muscular activity of the arm was recorded via surface electromyography (sEMG) electrodes. Contrary to prior work, asking amputees to perform “movements without movement” (Raffin et al., 2012b) allows us to investigate to which extent the amputees' eye-hand coordination has changed as a result of the amputation, rather than due to difficulties controlling a prosthesis. Given the similarity of movements executed with the phantom limb compared with those executed with intact limb (Raffin et al., 2012a,b), we also expect the eye-hand coordination of movements involving the missing limb to be highly similar to those involving the intact limb. This “ideal” setting does not imply that the results are not relevant for the prosthetic setting; the disruption of gaze strategies is actually characterized by a markedly longer reaching phase, while still maintaining the majority of the fixations on the target object (Sobuh et al., 2014; Hebert et al., 2019). The window of opportunity in the prosthetic setting is therefore expected to be considerably longer than the one we identify here.

The total size of the dataset exceeds 70 h of video, which is far too large to be analyzed and annotated manually within reasonable time. However, quantifying the distances between gaze point, target object, and the forearm is of fundamental importance for the present study. We therefore employed state-of-the-art deep learning techniques to automatically detect and segment all objects of interest in all videos. This procedure consisted of an efficient method to collect representative training data and the subsequent finetuning of a pretrained object detector to this data. A beneficial side-effect of detecting object locations in the video is that we can reliably determine fixations even in the presence of head movements.

In the following, we describe the dataset and the methods employed in the analysis in section 2. In section 3, we then present the results of our analysis, which are discussed more thoroughly in section 4. Finally, we conclude and summarize the paper in section 5.

## 2. Materials and Methods

To investigate the visuomotor behavior during manipulation actions we relied on a large, recently acquired dataset. In the following, we describe how the data were used in the context of the present study. Due to the large amount of video data contained in this dataset, we devised a procedure to automatically detect and segment all objects of interest via deep learning. This procedure is outlined and we formulate how the resulting segmentation masks were used to determine distances.

### 2.1. MeganePro Dataset

The MeganePro dataset was acquired with the aim of investigating the use of gaze and visual information to improve prosthetic control (Cognolato et al., 2019). It contains data of 15 transradial amputees [13 M, 2 F; age: (47.13 ± 14.16) years] and a frequency matched control group of 30 able-bodied subjects [27 M, 3 F; age: (46.63 ± 15.11) years] who performed grasps and manipulation tasks with a variety of household items. The gaze data for one of the amputated subjects was unreliable due to strabismus; this subject was therefore excluded from our analyses. The characteristics of the remaining amputated subjects is shown in Table 1, including information on the amputation and prosthetic use. All of them reported to experience phantom limb sensations, but only 12 had some voluntary control over the phantom limb.

**Table 1:** The characteristics of the amputated participants considered in the present study.

				**Amputation**				
**ID**	**Age**	**Gender**	**Handedness**	**Side**	**Cause**	**Years**	**Prosthesis**	**Limb [%]**
101	52	M	Right	Right	Electrocution	2	Cosmetic	60–80
102	39	M	Right	Right	Electrocution	4	Cosmetic	60–80
103	63	M	Ambidextrous	Right	Trauma	3	Myoelectric	60–80
104	49	M	Right	Right	Trauma	18	Myoelectric	80–100
105	73	M	Right	Right	Trauma	6	Body-powered	40–60
106	70	M	Left	Left	Trauma	5	Body-powered	80–100
107	36	M	Right	Left	Trauma	7	Body-powered	20–40
108	35	M	Right	Right	Trauma	9	Myoelectric	0–20
109	65	M	Right	Left	Trauma	1	Cosmetic	80–100
110	38	M	Right	Left	Trauma	14	Myoelectric	20–40
111	38	M	Right	Right	Trauma	10	Myoelectric	40–60
112	33	F	Right	Left	Oncological	13	Cosmetic	60–80
113	28	M	Right	Left	Trauma	7	Myoelectric	40–60
115	36	F	Right	Left	Burn	8	Cosmetic	n/a

*The table reports the ID of the subjects in the MeganePro dataset, their age, their gender, and their handedness. Among the clinical parameters we report the amputation side, its cause, the number of years since amputation, the type of prosthesis used, and the relative length of the residual limb with respect to the contralateral limb*.

**Figure 1:**
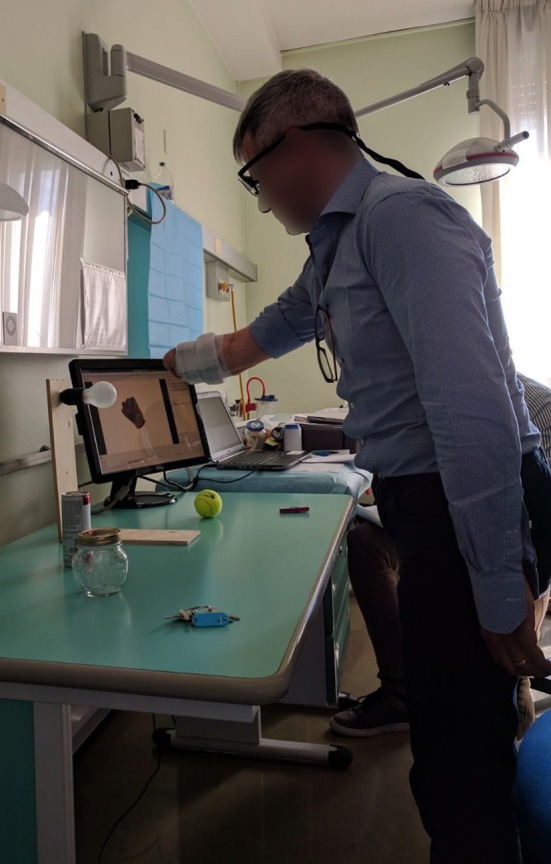
An overview of the experimental setup.

During the experiment, the subjects wore a Tobii Pro Glasses 2 eye-tracker (Tobii AB, Sweden) to record the gaze behavior, first person video, and angular velocities of the head. These glasses sample gaze and gaze-related information at 100 Hz, while the video is recorded with a 1920 px × 1080 px resolution at 25. On their forearm, they had 12 Delsys Trigno electrodes (Delsys Inc., USA) arranged in an array of eight equidistant electrodes at the height of the radiohumeral joint and four more electrodes in a second array 45 mm more distally. These electrodes record sEMG at 1926 Hz and contain an integrated three axes accelerometer that is sampled at 148 Hz. A picture showing the setup is shown in Figure 1.

The experiment consisted of repeatedly grasping or manipulating household items placed on a table in front of the subject. The pairing of grasps and objects was specifically chosen based (1) on the likelihood of their co-occurrence in ADLs and (2) to attain as much as possible a many-to-many relationship between grasps and objects. In the first part of the experiment, subjects just had to perform a “static” grasp on the object without any manipulation, hold it for a few seconds and then return to a rest posture when instructed. The amputated subjects were asked to attempt to execute the action as naturally as possible “as if their missing limb were still there,” rather than just imagining it, to elicit activation of the remaining muscles in their residual limb. Each of the grasps in Table 2 and its three associated objects were first introduced via a video, after which the subjects were instructed vocally to grasp each object four times while seated and then another four times while standing. The order in which the objects appeared in each repetition block was randomized to avoid habituation. During the second part of the experiment, the same ten grasps were instead executed as part of a “functional” movement, as can be seen in Table 3. In this case, the movements were performed either seated or standing, depending on which position would seem more likely in real life.

**Table 2:** Overview of the static tasks.

**Grasp**	**Object**		
Medium wrap	Bottle 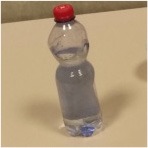	Door handle 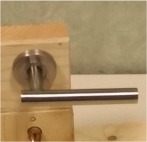	Can 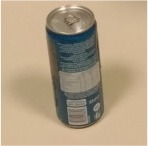
Lateral	Mug 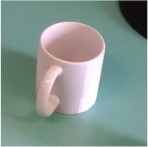	Key 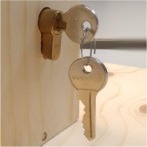	Pencilcase 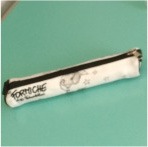
Parallel extension	Plate 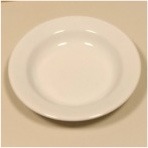	Book 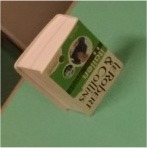	Drawer 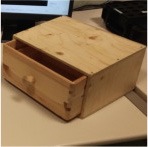
Tripod grasp	Bottle 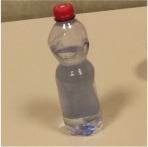	Mug 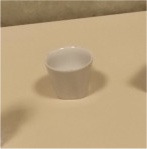	Drawer 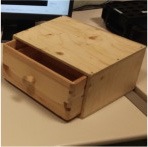
Power sphere	Ball 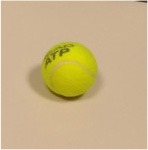	Bulb 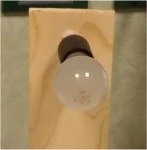	Key 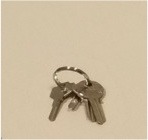
Precision disk	Jar 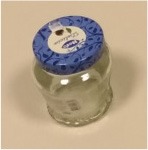	Bulb 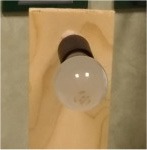	Ball 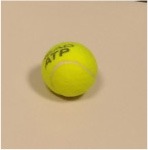
Prismatic pinch	Clothespin 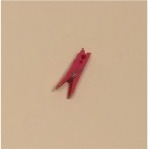	Key 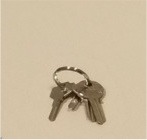	Can 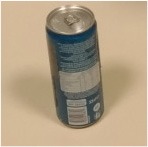
Index finger extension	Remote 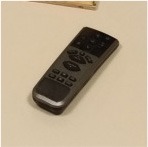	Knife 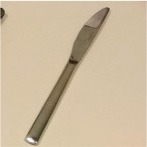	Fork 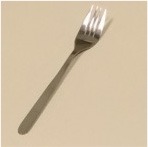
Adducted thumb	Screwdriver 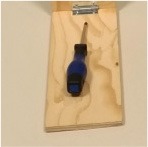	Remote 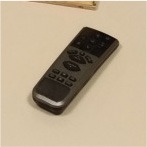	Wrench 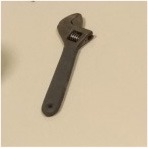
Prismatic four finger	Knife 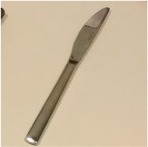	Fork 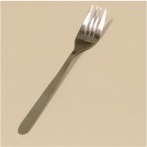	Wrench 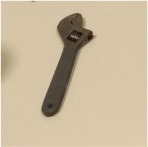

*For each row, the grasp and the associated objects are indicated. The subjects were asked to grasp each object with the given hand configuration while both seated and standing*.

**Table 3:** Overview of the functional tasks in the second part of the MeganePro dataset.

**Grasp**	**Vocal instruction**	**Position**	**Category**
Medium wrap	Drink from the **can**	Standing	Lifting
	Open and close the **door handle**		In place
Lateral	Turn the **key** in the lock	Standing	In place
	Open and close the **pencil case**		In place
Parallel extension	Lift the **plate**	Standing	Lifting
	Lift the **book**		Lifting
Tripod grasp	Open and close the cap of the **bottle**	Standing	In place
	Open and close the **drawer**		In place
Power sphere	Move the **ball** to the right and back	Standing	Displacement
	Move the **keys** forwards and backwards		Displacement
Precision disk	Open and close the lid of **jar**	Seated	In place
	Screw and unscrew the **light bulb**		In place
Prismatic pinch	Squeeze the **clothespin**	Seated	In place
	Move the **keys** forwards and backwards		Displacement
Index finger extension	Press a button on the **remote control**	Seated	In place
	Cut bread with the **knife**		In place
Adducted thumb	Turn the **screwdriver**	Seated	In place
	Move the **wrench** to the right and back		Displacement
Prismatic four finger	Move the **knife** forwards and backwards	Seated	Displacement
	Move the **fork** to the right and back		Displacement

*The vocal instruction in English indicates the task that had to be performed for each object-grasp pair, while the position denotes whether the subject performed the task while seated or standing. The last column indicates the movement category as per the description in section 3.3*.

Given the scope of the present paper, we only use sEMG from the second and seventh electrode, which were placed approximately on the extensor and flexor digitorum superficialis. Besides having relatively high activations, these electrodes also indicate roughly whether the hand was opening or closing. To aid visualization, both channels were rectified with a moving root mean square with a window-length of 29 ms (i.e., 57 samples) (Merletti, 1999). With respect to accelerometry, we note that the accelerations of all electrodes were highly correlated due to their positioning around the forearm. We therefore use accelerations only from the first electrode and normalize them with respect to the inertial frame of the initial position in each trial (Tundo et al., 2013).

### 2.2. Gaze Velocity

A common method to classify gaze events in fixations and saccades is based on the evaluation of the angular gaze velocity (Salvucci and Goldberg, 2000). Given two consecutive 3-dimensional gaze vectors ***g***_*i*−1_ and ***g***_*i*_, the angular difference between them can easily be calculated by means of their dot product (Duchowski, 2007)

(1) $$\begin{array}{r} \alpha_{i}=\arccos\left(\frac{g_{i}\cdot g_{i-1}}{∥g_{i}∥∥g_{i-1}∥}\right),\;\forall \text{i}\in \left\{2,\ldots ,N\right\}. \end{array}$$

An approximation of the instantaneous gaze velocity at time *t*_*i*_ then follows as

(2) $$\begin{array}{r} v_{i}=\frac{\alpha_{i}}{t_{i}-t_{i-1}},\;\forall \text{i}\in \left\{2,\ldots ,N\right\}. \end{array}$$

Although the Tobii glasses provide a unit gaze vector for both eyes, we instead use the gaze point in world coordinates to estimate the common angle of the eyes. These world coordinates had fewer missing data and were slightly cleaner in practice due to onboard processing. They are however relative to the position of the scene camera rather than the eyes. Since this camera is located on top of the frame of the glasses, this may lead to some inaccuracy at small gaze distances. We therefore map the gaze points to a coordinate system that is centered between the left and right pupils

(3) $$\begin{array}{r} {\hat{g}}_{i}=g_{i}-{\bar{p}}_{i},\;\forall \text{i}\in \left\{1,\ldots ,N\right\}, \end{array}$$

where ${\bar{p}}_{i}$ is the average of the left and right pupil locations relative to the scene camera. To limit the impact of missing data for the pupils, we linearly interpolated gaps shorter than 0.075 s (Olsen and Matos, 2012).

### 2.3. Object Detection and Segmentation

To determine whether the subject is fixating the target object at any given time, we need a precise segmentation of this latter object throughout the exercise. Since the videos for each subject totaled around 90 min or 135 000 frames, this would be very time consuming to annotate manually. We therefore employed a deep learning algorithm to automatically segment and classify all instances of our objects of interest (see Table 2). Finetuning this algorithm to our data still required at least a few dozen segmentations per object class. Rather than creating these manually, we instead used a second deep learning algorithm to facilitate the creation of this dataset.

#### 2.3.1. Creation of the Training Dataset

SiamMask is a recently proposed method for object tracking and semi-supervised video object segmentation (Wang et al., 2019). By marking just a bounding box around an object in one frame, this deep convolutional algorithm (1) segments the object from the background and (2) tracks it in the following frames in a video sequence. Although it may seem tempting to run this algorithm on an entire video annotating each object only at its first occurrence, in practice the object tracking does not work reliably on such long time scales. We therefore used this method to amplify our manual annotations; with just a single bounding box annotation per object, we obtain 10 to 20 times as many binary segmentation masks for our training set.

**Figure 2:**
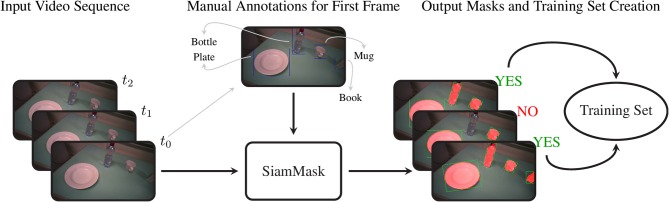
The procedure to acquire the training set of segmentation masks. We first select an arbitrary frame from a video and annotate each object with its bounding box and object identity. This information is passed to SiamMask, which produces segmentation masks for this initial frame and the subsequent frames in the video sequence. At each frame, the user can choose whether or not to include the frame and its segmentations in the training set or to move to a new initial frame.

For our approach, we embedded the official implementation of SiamMask^1^ with a default ResNet-50 backend in a custom application. This software allows the user to select a frame in a video and to annotate several objects with their bounding box and their class identity. Based on this initialization, SiamMask processes the initial frame and subsequent frames one by one. At each frame, the output is presented to the user for validation, who can either accept or refuse the proposed segmentation. This procedure is shown schematically in Figure 2. In practice, we accepted sequences up to about 15 frames. Applying this procedure repeatedly, we processed in total 2,422 frames with 11,726 segmented object instances chosen from 15 subjects. To include as much variability as possible in our dataset, we captured the objects from different perspectives, with different backgrounds, and while partially occluded. Furthermore, besides the eighteen objects in Table 2, we also included segmentations for a “person” class, which is primarily used to detect the subject's own limb.

#### 2.3.2. Training and Inference of Mask R-CNN

**Figure 3:**
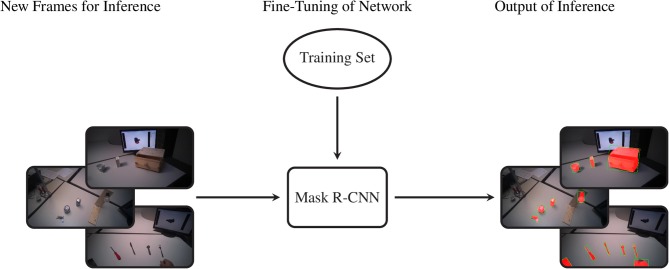
The procedure to segment the entire dataset. By means of the previously selected training set we fine-tune the Mask R-CNN model. Later we feed the network with new frames and it provides the segmented object instances as output.

The data we acquired in this manner were used to train Mask R-CNN on our objects of interest. This method detects and segments all instances of the known objects in an image (He et al., 2017). Rather than training a model “from scratch,” we bootstrapped from a model that was supplied with the implementation of Mask R-CNN by Massa and Girshick (2018). This model used a relatively standard ResNet-50-FPN backbone (Lin et al., 2017) and was pretrained on the COCO dataset (Lin et al., 2014), a large scale generic dataset for object detection, segmentation, and classification. As is common with finetuning, we replaced the final classification layer of the model with a random initialization and then performed additional training iterations with a reduced learning rate of 0.0025 to tailor the model to our custom dataset. The data of ten subjects were used for training, while the validation set consisted of the data of the remaining five subjects, which were chosen to be as representative as possible for the entire dataset. We chose to use the model that minimized the loss on the validation set (i.e., early stopping), which was obtained after just 4,000 iterations^2^. The performance of this model is compared in Table 4 with the average precision (AP) metrics of the pretrained model on the original COCO dataset. Note that due to the limited domain of our dataset and the smaller number of classes our performance compares favorably to the larger COCO dataset. After training, we employed the model in inference mode to detect and segment objects in all videos of all subjects, as shown graphically in Figure 3.

**Table 4:** Comparison of Mask R-CNN's detection accuracy on the COCO dataset and the accuracy of our finetuned model on the MeganePro dataset.

**Dataset**	**AP[%]**	**AP50 [%]**	**AP75 [%]**	**Source**
MeganePro	77.5	92.7	87.6	This work
COCO	33.6	55.2	35.3	He et al., 2017

*The AP is the average precision over Intersection over Union (IoU) from 0.5 to 0.95 evaluated at steps of 0.05. AP50 and AP75 represent the average precision when the threshold of is 0.5 or 0.75. A detailed description of these metrics can be found on the website of the COCO dataset (http://cocodataset.org)*.

#### 2.3.3. Distances

The segmentation masks for all videos were stored to disk and then combined with the gaze data to calculate various distances. In the following, we restrict ourselves to segmentations that were recognized with a certainty score of at least 0.8. The distances that are of interest for our analyses are the following.

- The *gaze-target* distance, which is the distance between the gaze point in frame coordinates and the target object for a grasp trial, if visible in the frame. If multiple instances of the same target class were recognized, then we chose the largest in terms of area.
- The *gaze-limb* distance denotes the distance between the gaze point and the hand or residual limb of the participant, if visible. We only consider instances identified as “human” that fall in the lower half of the image frame and again prefer the largest one.
- When applicable, the *limb-target* distance indicates the distance between the subject's hand or residual limb and the target object, as defined in the previous two distances.

Note that with the term “distance” we intend the minimum Euclidean distance in pixels between a point and the contour of a binary mask or between the contours of two binary masks. If these overlap, then the distance is 0. Note that with the scene camera of the Tobii glasses we find that 1 px ≃ 0.72 mm at a typical manipulation distance of 0.8 m.

### 2.4. Events

The profile of these distances and the modalities described previously were used to determine the timing of visuomotor events with respect to the stimulus, such as the first fixation on the target object or the onset of the arm movement. These events allow us to quantitatively describe the time interval between the activation of the eyes, head, and limb. The analysis window for each trial ranges from 2 s before until 2.5 s after the end of the corresponding vocal instruction with a resolution of 20 ms. We define the following events.

- The first *fixation* is defined as the first of at least two successive samples where the *gaze-target* distance is <20 px. This threshold was chosen to accommodate for some systematic error in the gaze tracking and is roughly twice the average gaze tracking accuracy (Cognolato et al., 2019). The requirement for two successive samples that fall below the threshold is to ignore occasional outliers.
- The *saccade* to the target object is assumed to initiate at the last sample where the gaze velocity was <70°/s (Komogortsev et al., 2010), starting from 500 ms prior to the target *fixation*. This definition in terms of the last preceding fixation rather than the first saccade makes it robust against missing data from the eye tracker during saccades. Furthermore, we require this saccade to start from a *gaze-target* distance of at least 100 px to avoid occasional trials where the subject was already fixating the target object.
- The start of the *head* movement is defined as the first of two successive samples where the Euclidean norm of the angular velocity vector of the Tobii glasses exceeds 12°/s. This threshold was chosen manually to be insensitive to systematic errors in the measurements of the gyroscope in the Tobii glasses.
- The movement of the *arm* starts at the first of two successive samples where the Euclidean norm of the three-axis accelerations exceeds 0.07 g. Also in this case the threshold was tuned manually to be insensitive to the baseline level of noise of the accelerometers.
- The activation of the *forearm muscles* starts when either of the myoelectric signals exceeds 4 times its baseline level for two successive samples. This baseline level is taken as the average activation in the rest period from 2 s to 1 s before the vocal instruction ended.
- Finally, the first *grasp* occurs when there are two successive samples where the *limb-target* distance is <5 px. This threshold was chosen to allow for a small error margin in the detected segmentation masks.

Whenever the conditions for an event were not satisfied it was marked as missing for the corresponding trial. Furthermore, we invalidate all events that were found within the first 100 ms of the analysis window, as it implies that the subject was not in a rest position or was already fixating the target object.

## 3. Results

In the following, we analyze the eye-hand coordination of the subjects in response to the grasp stimulus during the reach-to-grasp and manipulation phases. In other words, we relate movement of the eyes and head with that of the forearm. Before moving to these analyses, we verified that subjects effectively looked at the target object during a grasp trial. Thanks to the deep learning approach described previously, we determined that in 95.9% of the trials the *gaze-target* distance was <20 px at least once. Manual evaluation of the remaining 4.1% of the trials revealed that these were caused by a low accuracy of the gaze tracking that exceeded our threshold rather than lack of subject engagement.

### 3.1. Statistical Analysis

The first objective in this paper is to determine the window of opportunity in which gaze can provide useful information about an upcoming grasp. Table 5 shows that for intact subjects there is a median interval of 561 ms between the *fixation* event and the subsequent *grasp* event. The same interval increases to more than a second for amputated subjects, although this difference is because they did not physically interact with the objects and the *limb-target* distance therefore did not as often converge to within the 5 px threshold. Not surprisingly, a Kolmogorov-Smirnov test on the average interval per subject indicated that this difference between both subject groups was statistically significant. This is in contrast to the coordination between the initial saccade, the head, and the arm movements, for which we fail to find a significant difference between both groups. The saccade to the target object leads to its fixation in approximately^3^ 150 ms. Concurrently with the eyes, also the head starts to move. This head movement is then followed by acceleration of the arm around 130 ms later. In intact subjects, the activation of the forearm muscles comes only 80 ms after the onset of the arm movement in the median case. This interval is more than half a second longer for amputated subjects and this difference is found to be statistically significant.

**Table 5:** Statistical description of the intervals in seconds between various events.

	**Intact**				**Amputated**				
**Interval**	**#**	**Q1**	**Med**.	**Q3**	**#**	**Q1**	**Med**.	**Q3**	**Significance**
fixation → grasp	8,144	0.321	0.561	0.842	1,942	0.581	1.042	1.644	KS = 0.724, *p* = 2.602 × 10^−5^
saccade → fixation	5,625	0.080	0.160	0.301	2,522	0.060	0.140	0.281	KS = 0.190, *p* = 0.811
saccade → head	5,419	−0.301	0.020	0.160	2,367	−0.461	−0.020	0.140	KS = 0.338, *p* = 0.173
head → arm	7,929	0.020	0.120	0.301	3,507	0.000	0.140	0.371	KS = 0.262, *p* = 0.447
arm → muscles	7,907	−0.020	0.080	0.401	3,576	0.200	0.581	1.042	KS = 0.829, *p* = 4.524 × 10^−7^

*The count refers to the number of trials where both events were recognized, out of a total of 9,703 trials for intact and 4,482 trials for amputated subjects, respectively. For the Kolmogorov-Smirnov test the intervals were averaged per subject to guarantee independent samples*.

### 3.2. Reach-to-Grasp Phase

**Figure 4:**
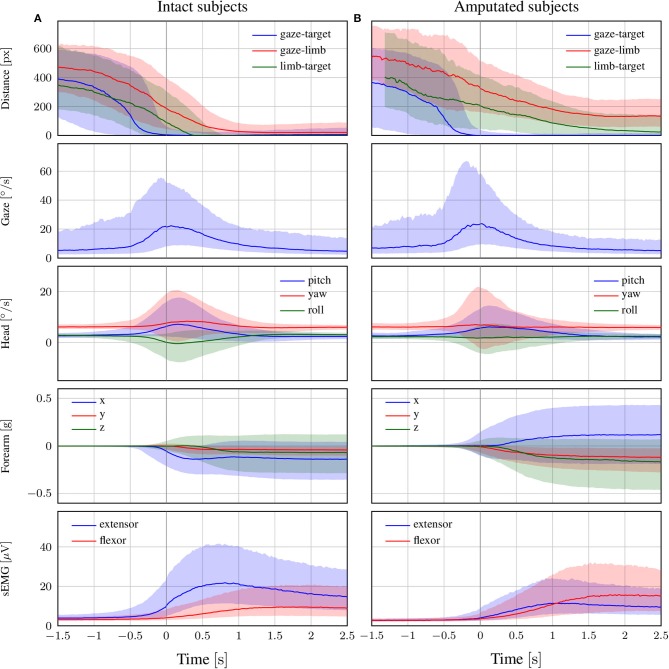
The trend of each modality in the reach-to-grasp phase for **(A)** intact and **(B)** amputated subjects. The zero corresponds to the end of the vocal instruction that indicated the required manipulation action. The solid line represents the median over all trials from all subjects, whereas the shaded areas indicate the 25th and 75th percentiles. Segments with more than 90% missing data were omitted.

The coordination during the reaching phase of all “static” and “functional” grasps is visualized in Figure 4 for both intact and amputated participants. Whereas the previous statistical analysis was intended to provide a quantitative assessment of the relative timings in eye-hand coordination, this figure instead complements those numbers by demonstrating how this coordination evolves over time. It does so by showing the median and quartiles of the distribution over all trials from all subjects in either group from 1.5 s before to 2.5 s after the conclusion of the vocal instruction. For both types of subjects, we observe an increase in gaze velocity from −0.5 s to 1 s. This increase also marks a sharp decrease in the distance between the gaze and the target object, which leads to a fixation soon after. From this moment on, the subjects retain their fixation on the object of interest. Based on the median profiles, we see again that the onset of the head movement starts around the same time as the eye movement and continues for 1.5 s.

The delay of the arm movement with respect to the eyes is slightly larger for amputated subjects, as shown by the median profile of the forearm's acceleration in Figure 4. Shortly after the arm starts to move, we also observe an increase in sEMG activity, with initially an emphasis on the extensor and later on the flexor. For able-bodied subjects, the profile of the *limb-target* distance confirms our earlier finding that the limb arrives at the object 500 ms after its fixation. Although this result is not directly comparable with that for amputated subjects, we observe that the convergence between their residual limb and the target object appears more gradual and is characterized by a much larger variability.

A noteworthy observation is that the activation of the eyes always preceded the end of the vocal stimulus. The reason is that subjects could typically deduce the target object already before the end of the instruction. This does not affect our results, since we are interested in the relative delay between eyes, head, and forearm rather than reaction times to the stimulus. The differences in reaction time to the vocal instructions do increase however the dispersion of the distributions. We also note that the relative contribution among the three axes of the acceleration profile differs between able-bodied and amputated subjects. The reason is that we normalized this profile with respect to the initial position of the forearm, which is typically different for both types of subjects. In the present study, we use accelerometry to determine when the arm starts to move and rely on the limb-target distance to measure its convergence to the target object.

### 3.3. Manipulation Phase

**Figure 5:**
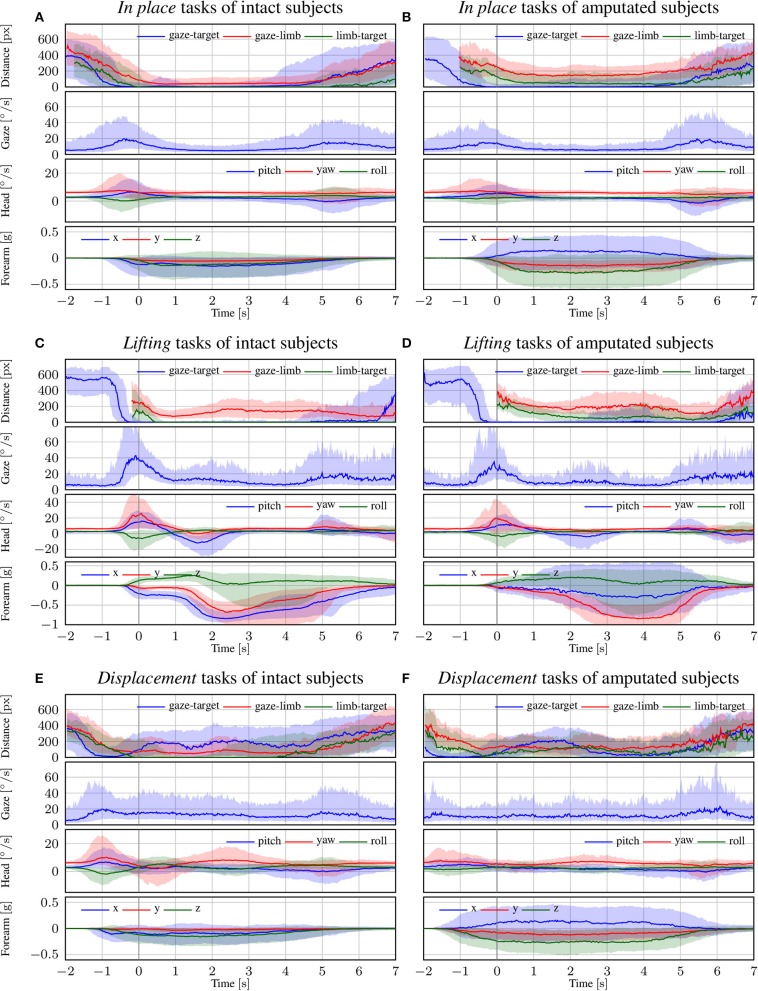
The trend of each modality for intact and amputated subjects for the **(A,B)** *in place*, **(C,D)** *lifting*, and **(E,F)** *displacement* functional tasks. The zero corresponds to the end of the vocal instruction that indicated the required manipulation action. The solid line represents the median over all trials from all subjects, whereas the shaded areas indicate the 25th and 75th percentiles. Segments with more than 90% missing data were omitted.

In Figure 5, we focus on the behavior of intact and amputated subjects during the functional tasks to further investigate the similarities in gaze strategy. These figures start from 2 s before the vocal instruction until 7 s after, which is enough to cover the entire manipulation action. We group the MeganePro movements into three categories based on the type of task and the associated visual behavior, as shown in Table 3. These categories are *in place* manipulation actions, *lifting* actions, and finally *displacement* actions.

#### 3.3.1. In Place Actions

**Figure 6:**
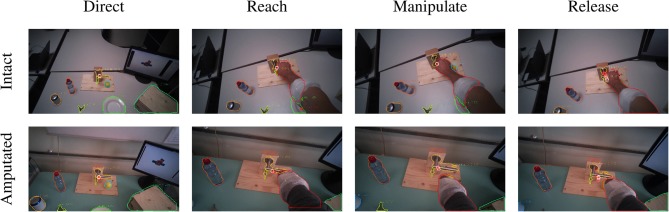
Example of the visuomotor behavior of an intact (first row) and an amputated (second row) participant while opening a door handle. The gaze trail is represented by the circles from the current gaze position (red) to ten samples later (white). Both subject groups direct the gaze on the object during the reaching phase (first column). The eyes then remain focused on the target object during the grasping and manipulation phases (second and third columns). In both cases, the motor behavior of the arm is similar for intact and amputated subjects. During the release phase the gaze shifts away from the object (fourth column).

The *in place* actions concern manipulation tasks that do not require moving the object, like opening an object, cutting bread, or pressing a button of the remote control. The aggregated profiles of all modalities for these actions are shown in Figure 5A for able-bodied subjects and in Figure 5B for amputees. During this type of action, the gaze remains fixed on the target object throughout the entire duration of the manipulation, as can also be seen in the example in Figure 6 that overlays gaze and object segmentations on representative frames of the first person video. As expected, the hand remains on the target for the entire duration in case of able-bodied subjects, whereas for amputees there remains a constant subject-dependent distance between the residual limb and the target. Head movements are limited to the initial reach-to-grasp phase to center the object in the field of view, after which the head remains fixed until the end of the manipulation.

#### 3.3.2. Lifting Actions

**Figure 7:**
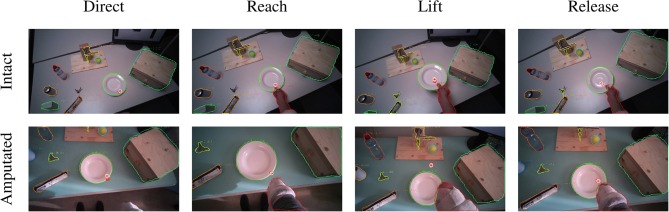
Example of the visuomotor behavior of an intact (first row) and an amputated (second row) participant lifting a plate. The gaze trail is represented by the circles from the current gaze position (red) to ten samples later (white). The eyes focus on the manipulation point to plan the hand's approach (first and second columns). During the lifting phase, the eyes move away from the reaching point and the amputee's gaze even exceeds the mask boundary of the plate (third column). The object is fixated again during the release (fourth column).

The second group is composed of *lifting* actions, in which the subject was required to lift an object up and then place it back in its initial position. As can be seen in Figures 5C,D, also in this case the gaze anticipates head and forearm movement. More interestingly, we see a clear movement in the pitch orientation of the head. Since these actions are executed while standing, the subjects first lower their head to locate the target object on the table. Then, when they have located and grasped the object, they raise their head again with a peak pitch velocity at 1.7 s for able-bodied subjects and slightly later for amputated subjects. This head movement coincides with a modestly increased gaze velocity and is due to the tracking motion of the lifting action. In some cases, this tracking strategy even caused an amputated subject's *gaze-target* distance to increase, as can also be seen in the example in Figure 7. Finally, the subjects lower their head again when tracking the release of the object at the end of the trial.

#### 3.3.3. Displacement Actions

**Figure 8:**
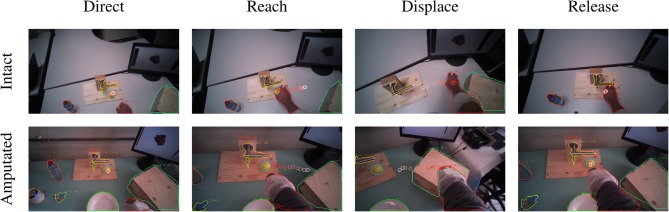
Example of the visuomotor behavior of an intact (first row) and an amputated (second row) participant while moving a ball. The gaze trail is represented by the circles from the current gaze position (red) to ten samples later (white). The gaze focuses on the object until the hand's arrival (first column), when the grasping phase begins the eyes shift away toward the destination (second column). When the hand reaches the destination the gaze shifts back to the initial location (third column) to release the target (fourth column).

The final category are the so-called *displacement* actions. During these tasks, the subjects had to grasp the objects, move them horizontally to another position, and then place them back in the initial position. We note that the gaze and motor behavior starts earlier with respect to the vocal instruction. For this category of tasks, the name of the object happens to appear at the beginning of the instruction (see Table 3), thus allowing subjects to initiate the task early. For intact subjects, we see in Figure 5E that 200 ms before the hand reaches the object the *gaze-target* distance starts to increase again. The gaze, in this case, shifts already to the destination position for the displacement action, as demonstrated in the second panel in Figure 8. Although less pronounced, the same pattern repeats itself at around 1.5 s when the subject initiates the return movement. The profiles for the amputated subjects in Figure 5F show different behavior, with an overall increase in *gaze-target* distance throughout the entire duration of the movement. As intact subjects did, their gaze anticipates the path of the hand rather than the path of the object, which is not physically displaced. This strategy is demonstrated clearly in the bottom row of Figure 8.

## 4. Discussion

The objective of this paper was to determine the window of opportunity for exploiting gaze as contextual information in decoding the grasp intent of amputees. A related question was to which extent the natural gaze strategies of amputees and able-bodied subjects were similar. After comparing our results with related work, we discuss both topics. Finally, we argue for the use of recent developments in deep learning in the analysis of large-scale visuomotor studies.

### 4.1. Visuomotor Strategy and Comparison With Related Work

In section 3.2, we presented the results of eye, head, and limb coordination during reaching and grasping. The eyes are the first to react to the vocal stimulus by exhibiting an increasing saccade-related activity, leading to a fixation on the target in about 150 ms. When the eyes start moving, also the head follows almost immediately. Such short delays between movement of the eyes and the head have been reported in the literature, ranging from 10 ms to 100 ms during a block-copying task (Smeets et al., 1996) or in reaction to visual stimuli (Goldring et al., 1996; Di Cesare et al., 2013). This behavior is however strongly dependent on the experimental setting and even small variations therein can change the outcome. For instance, Pelz et al. (2001) found that depending on the exercise's instruction the head may both precede (by about 200 ms) or follow the eyes (by about 50 ms) in the same block-copying task.

After the activation of the eyes and the head we observe the movement onset of the arm 130 ms later. Similar values ranging from 170 ms to 300 ms were also reported by Smeets et al. (1996) and Pelz et al. (2001) in a block-copying task and by Belardinelli et al. (2016) in a pick and place task. Land et al. (1999) instead found a median delay of 0.56 s during a tea-making activity. Rather than movement onset, the time the hand takes to reach the target is more interesting for our scope. For the intact subjects, the hand typically starts to occlude the target object around 500 ms after the first fixation. Although occlusion does not necessarily already imply a completed grasp, especially given the first person perspective, we do expect the grasp to follow not much later. These results confirm that visual attention on objects anticipates manipulation. In previous studies concerning displacements (Johansson et al., 2001; Belardinelli et al., 2016; Lavoie et al., 2018) and grasping activities (Brouwer et al., 2009), a variable delay ranging from 0.53 s to 1.3 s was found between the eye and hand. Also in these cases, the exact value of the delay depends on the characteristics of the experiment.

In section 3.3, we concentrated on the visuomotor strategy adopted by amputated and able-bodied subjects to interact with the objects during three groups of functional tasks. We can characterize the strategies associated with these groups in terms of the types of fixations defined by Land et al. (1999) and Land and Hayhoe (2001), namely *locating, directing, guiding*, and *checking*. A fixation to *locate* is typically done at the beginning of an action, to mentally map the location of objects that is to be used. Instead, a fixation to *direct* is meant to detect an object that will be used immediately after. Fixations to *guide* are usually multiple and occur when the gaze shifts among two or more objects that are approaching each other. Finally, there are long *checking* fixations to monitor the state of an action waiting for its completion.

The visual strategy of the *in place* actions is relatively straightforward. In these tasks, subjects initiate with a fixation to *direct* the attention to the target object. Subsequently, their fixation remains on the manipulated object to *check* the correct execution of the task. Note that this visual attention seems focused on the target object rather than the subject's hand, as can be seen comparing the *gaze-target* and *gaze-limb* distances in Figures 5A,B. Indeed, Land et al. (1999) noted that the hands themselves are rarely fixated.

Also the *lifting* actions start with a *directing* fixation to locate the object of interest. However, whereas the initial fixation is focused on the intended grasp location (cf. the left column in Figure 7), the gaze shifts upwards when the hand has grasped the object. This coincides with the transition from the *directing* fixation to visually *checking* the lifting action. This is in line with observations by Voudouris et al. (2018), who noted that people may fixate higher when grasping and lifting an object to direct their gaze to where the object will be in the future.

Finally, *displacement* actions are the ones most investigated in the literature. Previous studies on pick and place tasks (Belardinelli et al., 2016; Lavoie et al., 2018) and on the block-copying task (Smeets et al., 1996; Pelz et al., 2001) fall in this category. In this case, we observe in Figure 5E that the *gaze-target* and *gaze-limb* distances have three minima for intact subjects, namely at the initial pick-up, the destination, and at the release again at the initial position. All three minima indicate fixations that are meant to *direct* the approach of the hand, either for (1) grasping the object, (2) displacing it, or finally (3) releasing it. This behavior can clearly be seen for both intact as well as amputated subjects in the example in Figure 8. We also notice that the eyes did not wait for the completion of the pick-up action, moving instead toward the position of the destination around 200 ms in advance. This proactive role of the eyes was highlighted by Land et al. (1999), who measured the gaze moving on to the next object between 0 s to 1 s before the current object manipulation was terminated. Also Pelz et al. (2001) observed the eyes departing from the target object 100 ms to 150 ms before the arrival of the hand.

### 4.2. Comparison Between Intact and Amputated Subjects

One of the aims of this work was to understand if a transradial amputation has introduced important changes in the visuomotor behavior of amputees. During the reach-to-grasp phase, the overall behavior of intact and amputated subjects is comparable. Even if the coordination timeline between eyes, head, and limb is similar, there are some minor discrepancies between the two groups. The main observed difference concerns the delayed activation of the forearm muscles during the reaching phase for amputated subjects, which was found to be statistically significant. Similarly, during the lifting tasks we noted slower pitch movements of the head. It is likely that some subjects interpreted the instruction to perform the grasp with their missing limb by activating their phantom limb. Such movements executed with the phantom limb are known to be slower than those executed with the intact hand (Raffin et al., 2012b; De Graaf et al., 2016).

Throughout the manipulation phase, we observe a striking similarity in visuomotor strategy between the amputated subjects and the control group. The differences that we noted in the results are not due to an alternative gaze strategy, but rather because the objects were not physically moved during the interaction. For instance, in the *lifting* task visualized in Figure 5D we saw an increase in *gaze-target* distance in the range from 2 s to 5 s. This increase was due to an upward shift in the gaze location to track where the object would have been if it had been lifted for real. Similarly, during the displacement task in Figure 5F we do not observe a minimum in *gaze-target* distance at around 1.5 s, as was the case for intact subjects (see Figure 5E). Instead, around the same time we observe a peak for the amputated subjects, solely because the target object is still at its original position whereas their gaze has shifted to the intermediate destination. The examples for these gaze strategies in Figures 7, 8 demonstrate how similar intact and amputated subjects behaved.

It would be interesting to understand how these results relate to the disrupted eye-hand coordination when using a prosthetic device. Previous studies (Bouwsema et al., 2012; Sobuh et al., 2014; Parr et al., 2018) have underlined that prosthetic users are more fixated on guiding the current manipulation, rather than planning the follow-up action. This behavior is most likely caused by the fact that amputated people rely almost exclusively on visual feedback. However, since only a small number of subjects were engaged in the previous studies more research will be needed to fully understand the disruption of the visuomotor strategy. In particular, whether or not this strategy improves when the user develops trust in the prosthesis (Chadwell et al., 2016) merits attention. Another equally interesting question is to which extent the proactive gaze behavior can be restored by integrating tactile or proprioceptive feedback in the prosthesis (Cipriani et al., 2011; Marasco et al., 2018; Markovic et al., 2018, among others).

### 4.3. Integration of Vision in Prostheses to Improve Intent Recognition

The estimated time interval from *fixation* to *grasp* in section 3.1 shows that the window of opportunity is 500 ms for intact subjects. This interval cannot be accurately determined for amputated subjects, as they executed the movement with their missing limb and therefore lacked physical contact with the target object. Although Figure 4B suggests that this window will at least be as long for amputated users, one may argue that this result is not representative for movements performed with a prosthesis. However, previous studies showed without exception that prosthetic users still fixate the target object for the majority of the reaching phase (Bouwsema et al., 2012; Sobuh et al., 2014; Chadwell et al., 2016; Hebert et al., 2019; Parr et al., 2019), albeit alternating it more often with fixations on the hand (i.e., the “switching” strategy). Moreover, this reaching phase may actually take more than twice as long as compared to the same movement performed with the anatomical limb (Sobuh et al., 2014; Hebert et al., 2019). These findings suggest that the target object will still be fixated proactively by a prosthetic user and that the window of opportunity will more likely be longer than shorter.

Exploiting this anticipatory gaze behavior is appealing because it comes naturally and therefore does not require specific attention from the user. The success of this approach relies however on the ability to distinguish informative fixations from those that are not necessarily related to any grasp intent. Gigli et al. (2018) attempted to address this problem by including the onset of the arm movement as an additional condition, which we have shown here to shorten the window of opportunity. Also the method that is used to detect fixations may shorten this window. Thanks to the frame-by-frame segmentations in the present study, we could accurately and instantaneously recognize object fixations by measuring the distance between the object's segmentation mask and the gaze point. In contrast, common fixation classifiers, such as (IVT) (Salvucci and Goldberg, 2000), define a fixation simply as the lack of eye movement. In reality, gaze shifts more commonly involve not only eye movement, but also head and sometimes even trunk movements (Morasso et al., 1973; Land, 2006). When the head moves, the optokinetic and vestibulo-ocular reflexes cause the eyes to counteract the head movement to maintain a stable gaze point (Lappe and Hoffmann, 2000). It is exactly due to such coordinated gaze movements that the initial object fixation in Figure 4 actually coincides with a *peak* in gaze velocity. The need to detect fixations as early as possible therefore implies a detection method that uses more information than eye movement alone. Whether this is best done by compensating for head movements (Kinsman et al., 2012; Larsson et al., 2014) or by comparing the visual object at the gaze point as in the present study is an open question.

A final consideration is regarding technical and practical concerns of a prosthetic solution that integrates eye tracking. Myoelectric control of prostheses has a long history and a solution that decodes natural muscle activations via pattern recognition is commercially available (Coapt, LLC, 2015). Tracking a user's gaze continuously and reliably in a variety of conditions will pose a bigger problem, however. The Tobii glasses used for the MeganePro dataset resulted in 10.7% of missing data on average, caused discomfort to the subjects after wearing them for about 2 h, and needed a battery replacement after 1.5 h to 2 h of continuous acquisition. Recent developments have seen considerable improvements however in terms of weight, cost, and aesthetic appeal (Pupil Labs GmbH, 2019).

### 4.4. Advantages of Deep Learning for the Automatic Analysis of Visual Behavior

Without the deep learning approach described in section 2.3 it would have been extremely labor intensive to analyze 70 h of video and data from 44 subjects. Manufacturers of eye-tracking devices often provide applications for semi-automatic analyses, but these do not allow the level of automation nor precision as the procedure described here. Although the object segmentations produced by Mask R-CNN were occasionally mistaken, the segmentations seen in the examples from Figures 6–8 are illustrative for the overall performance. It may easily be overlooked that data from research studies, such as the present, often contain much less visual variability than the datasets on which these algorithms are trained and evaluated. With minimal finetuning efforts, it is therefore likely to obtain levels of performance that considerably exceed those reported in the literature, as was seen in Table 4.

## 5. Conclusions

In this study, we analyzed the coordination of eye, head, and limb movements of amputated and able-bodied participants engaged in manipulation tasks of household objects. Our aim was to understand the anticipatory role of gaze in the visuomotor strategy and to determine whether this could potentially be used to aid in the grasp intent recognition for upper limb prostheses. We found that a fixation on the target object typically preceded the subsequent grasp by 500 ms in intact subjects and possibly longer for amputees. Moreover, the visuomotor strategies of amputees were similar to those of intact subjects both during the reach-to-grasp phase as well as during functional manipulation tasks. In future work, we aim to use the knowledge gained in this study to integrate vision with the (sEMG) modality to verify whether we can realize an effective improvement in recognizing grasp intentions during the reaching phase.

## Data Availability Statement

The datasets generated for this study are available on request to the corresponding author.

## Ethics Statement

The studies involving human participants were reviewed and approved by Ethics Commission of the canton of Valais in Switzerland and by the Ethics Commission of the Province of Padova in Italy. The patients/participants provided their written informed consent to participate in this study. Written informed consent was obtained from the individual(s) for the publication of any potentially identifiable images or data included in this article.

## Author Contributions

The consortium contributed the initial conception of the study. VG and AG contributed conception and design of the study. MC, GS, MA, and the consortium collected the dataset. VG devised and implemented the automated segmentation under the supervision of AG. VG collected the segmentation dataset. VG performed the visuomotor analyses under the supervision of AG. VG wrote the first draft of the manuscript. AG rewrote sections of the manuscript. All authors contributed to manuscript revision, read, and approved the submitted version.

### Conflict of Interest

The authors declare that the research was conducted in the absence of any commercial or financial relationships that could be construed as a potential conflict of interest.

## Footnotes

^1^https://github.com/foolwood/SiamMask

^2^The model is publicly available online (Gregori and Gijsberts, 2019).

^3^This is likely a slight overestimation, considering our definition of the *saccade* and missing values in the gaze data from the Tobii glasses.

## Consortium

Aside from the authors, the MeganePro consortium consists of Katia Giacomino and Anne-Gabrielle Mittaz Hager (University of Applied Sciences Western Switzerland “HES-SO Valais,” Leukerbad, Switzerland), Diego Faccio, Cesare Tiengo, and Franco Bassetto (Padova University Hospital, Padova, Italy), Barbara Caputo (Istituto Italiano di Tecnologia, Turin, Italy and Politecnico di Torino, Turin, Italy), Peter Brugger (University Hospital of Zurich, Zurich, Switzerland), and Henning Müller (University of Applied Sciences Western Switzerland “HES-SO Valais,” Sierre, Switzerland).

## Abbreviations

ADL: activity of daily living
sEMG: surface electromyography
IVT: Identification Velocity Threshold
FPN: Feature Pyramid Network
COCO: Common Objects in COntext
AP: average precision
IoU: Intersection over Union.
